# Creation of Molecular Gel Materials Using Polyrotaxane-Derived Polymeric Organogelator

**DOI:** 10.3390/gels9090730

**Published:** 2023-09-08

**Authors:** Yutaka Ohsedo, Tomoka Shinoda

**Affiliations:** 1 Division of Engineering, Faculty of Engineering, Nara Women’s University, Kitauoyahigashi-machi, Nara 630-8506, Japan; 2 Faculty of Human Life and Environment, Nara Women’s University, Kitauoyahigashi-machi, Nara 630-8506, Japan

**Keywords:** molecular gel, organogelator, polyrotaxane, thixotropic behavior, composite

## Abstract

Molecular gels, which are soft and flexible materials, are candidates for healthcare, cosmetic base, and electronic applications as new materials. In this study, a new polymeric organogelator bearing a polyrotaxane (**PR**) structure was developed and could induce the gelation of *N*′,*N*″-dimethylformamide (DMF), a known solvent for dissolving polymeric materials and salts. Furthermore, the resulting DMF molecular gels exhibited thixotropic properties, observed by the inversion method using vials, which are essential for gel spreading. The scanning electron microscopy of the xerogels suggested that the gel-forming ability and thixotropic property of gels were imparted by the network of the laminated aggregates of thin layer material similar to those of other gels made of clay materials. This thin layer material would be formed by the aggregation of polymeric organogelators. The dynamic viscoelasticity measurements of the obtained gels revealed the stability and pseudo-thixotropic behaviors of the obtained gels, as well as a specific concentration effect on the mechanical behavior of the gels attributed to the introduction of the **PR** structure. Additionally, the preparation of the polymer organogelator/polymer composites was investigated to improve the mechanical properties via the filler effect induced by the agglomerates of organogelator. Moreover, the tensile tests confirmed that the introduction of the gelator enhanced the mechanical properties of the composites.

## 1. Introduction

Molecular gels are material forms in which a solvent is retained by a network of low molecular weight gelators (LMWGs) or polymer gelators [1,2,3,4]. Regarding LMWGs, the building blocks of the network are the fibers that are formed by the self-assembled (supramolecular) layering of low molecular weight compounds through intermolecular interactions. Conversely, the building blocks of the network of polymeric gelators are the fibers that are formed by polymer assemblies [1]. As self-assembly induced by reversible intermolecular interactions represents a critical process in the formation of molecular gels, these gels are susceptible to changes in the external environment. Such changes account for external stimulus responsiveness during the formation of molecular gels, which are being developed for application as a variety of functional materials [5,6]. Other gel-like material forms include gel states comprising networks that are formed by chemical bonds (polymer gels) and those comprising network-like states formed by the “house of cards” structure of clay minerals (nanosheets), which are sheet-like inorganic compounds [7,8]. Regarding their advantages, molecular gels composed of LMWGs or polymer gelators can undergo diverse molecular designs and selections, and target molecular gel materials can be readily obtained by organic synthesis [1,2,3,4]. As the network that maintains the gel state of molecular gels comprises physical interactions that can be formed reversibly, i.e., physical crosslinking, its disintegration by an external mechanical force and its recovery with time are repeatedly observed, and this property is known as thixotropy [9,10,11,12,13]. Thixotropy indicates the spreading of materials by coating; it exists in soft materials with loose network structures, such as molecular gels and particulate systems. Currently, thixotropy is attracting attention as a required property of base materials for healthcare and cosmetic materials [12,13,14,15,16,17]. It is also crucial in device fabrication involving coating materials for electronic functional materials; therein, the thixotropy of the utilized gel (base material) is key to reducing viscosity during coating and retaining shape after coating [18,19]. The thixotropic property of the utilized ink substrate is key to ensuring viscosity reduction and shape retention during and after applications, respectively.

**Scheme 1:**
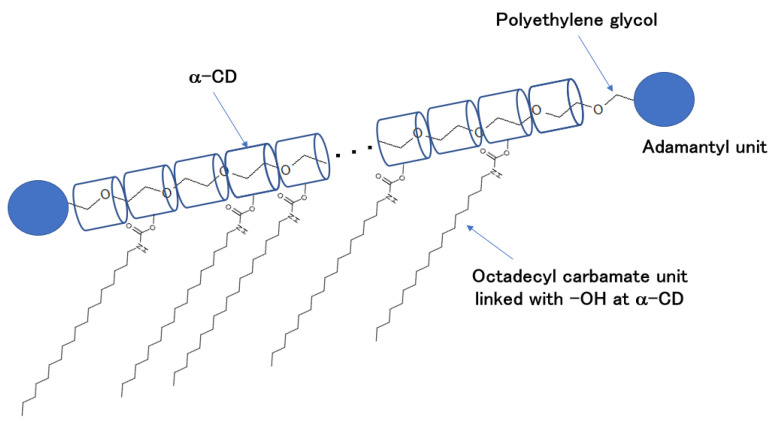
Schematic of the **PR**-derived polymeric organogelator (**PR-U**).

As a part of our research on the fabrication of new molecular gel materials, we have investigated the fabrication and application of new polymer gelators that can form thixotropic molecular gels. We have also investigated the properties of water-soluble aromatic polyamide polymers as polymer hydrogelators [20] and their application to organic–inorganic or organic–organic composites using thixotropic base materials [21,22,23,24]. In this study, we designed and synthesized a polymeric organogelator containing a polyrotaxane (**PR**) structure with an octadecyl carbamate unit (urethane unit, **U**), **PR-U**, and evaluated the mechanical properties of the obtained gel-like material by dynamic viscoelasticity measurements, as well as investigating the relationship between these properties and internal microstructure (Scheme 1). **PR**s [25,26,27,28] represent a group of inclusion compounds in which a polymer compound penetrates the vacancies of a cyclic compound, such as cyclodextrin, like a necklace; they are being actively studied as a new group of functional polymers. It has been demonstrated that the introduction of **PR** structures into polymers and gels facilitates the efficient dissipation of external mechanical forces and adds functionalities to gel materials [29,30,31,32,33]. The introduction of this structure as a unit of gelators may yield new polymer gelators that can form molecular gels characterized by the dissipation of external mechanical forces. In this study, we observed that the resulting molecular (organo)gel exhibited thixotropic properties. Next, we investigated the relationship between the internal microstructure and mechanical properties of the resulting gel-like materials. Additionally, the fabrication of polymer composites using this gelator as a filler agent was investigated using rubber bands (commercially available rubber materials) as the matrix material.

## 2. Results and Discussion

**Figure 1:**
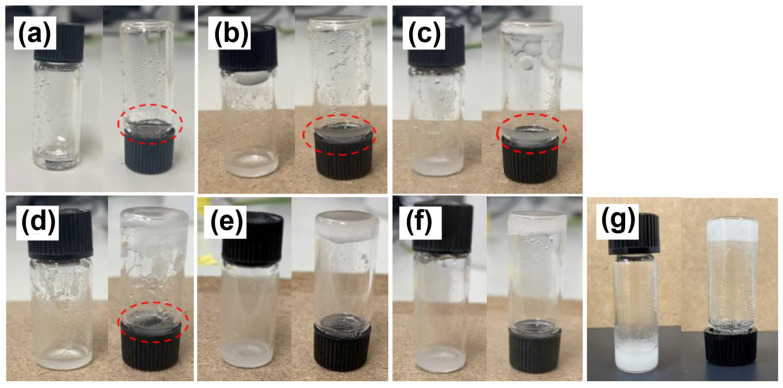
Gelation behaviors of **HP-PR** and **PR-U** in DMF: (**a**) 5.0 wt.% **HP-PR**, (**b**) 5.0 wt.% **PR-U-1**, (**c**) 5.0 wt.% **PR-U-2**, (**d**) 4.0 wt.% **PR-U-3**, (**e**) 4.5 wt.% **PR-U-3**, (**f**) 5.0 wt.% **PR-U-3**, and (**g**) 10.0 wt.% **PR-U-3** (a red circle indicates dropped gel).

The octadecyl carbamate-modified **PR**s, **PR-U**, were obtained from hydroxypropylated **PR**, **HP-PR** [34]. **HP-PR** with the inclusion rate of CD into the PEG chain of 22–25% based on the ethylene oxide moiety was obtained from adamantyl-capped **PR** comprising α-cyclodextrine (α-CD) and polyethylene glycol (PEG, molecular weight: 35,000) [35]. The ratios of the introduced **U** unit to –OH at position 6 of α-CD, as determined by proton nuclear magnetic resonance (^1^H-NMR) measurements with the results of elemental analysis, were **PR-U-1:** 16%, **PR-U-2:** 37%, and **PR-U-3**: 44% (these mean that the estimated introduction number of the octadecyl carbamate to **HP-PR** were 150 for **PR-U-1**, 349 for **PR-U-2**, and 410 for **PR-U-3**). The yields were obtained as white solids. The solubilities of the **PR-U** samples in organic solvents were also investigated. As they exhibited solubility when heated in *N*′,*N*″-dimethylformamide (DMF), which is highly soluble in a variety of organic compounds and inorganic salts, they were used in the subsequent experiments. Figure 1 shows the states of the mixed solution of DMF with **HP-PR** and **PR-Us** at different concentrations, as well as the gel-forming ability that was determined by inverting the vial. Figure 1b,c show that the mixture of DMF with **PR-U-1** and **PR-U-2** formed only a cloudy liquid and did not form a gel after standing for 30 min at 25 °C. Figure 1d–g show that the mixture of DMF and **PR-U-3** transformed into a cloudy liquid and cloudy gel at 4.0 wt.% and 5.0 wt.%, respectively, after standing at 25 °C. The formed gel did not pour out when the vial was turned upside down. However, the gel became uneven after ≥30 min, with solid and liquid portions, and was very loose and brittle. After the gel formation, the obtained gel transformed into a liquid when touched with a spatula; at 10.0 wt.%, the gel became cloudy and remained in the gel state without becoming liquid over time. When it was scooped with a spatula, we confirmed that it was a creamy soft gel. We observed that the **PR-U-3** gelled at >5.0 wt.%, which means the critical gel concentration of **PR-U-3** is 5.0 wt.%, and that 10.0 wt.% **PR-U-3** gelled, transforming into a creamy gel. Among the samples, the 10.0 wt.% **PR-U-3** maintained its gel state. Thus, **PR-U-3** was used as the gelling agent in the subsequent experiments.

**Figure 2:**
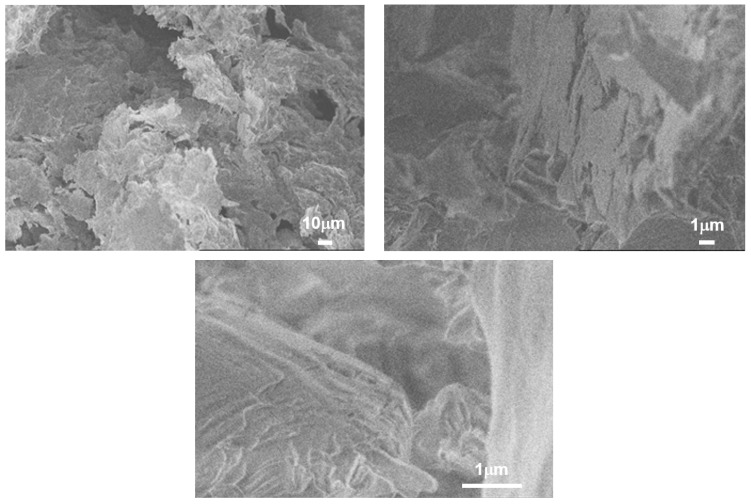
SEM images of the **PR-U-3** xerogel obtained by vacuum drying the 5.0 wt.% DMF gel.

Scanning electron microscopy (SEM) was used to observe the internal structure of the **PR-U-3** 5.0 wt.% DMF gel, which is the lowest gelling concentration (Figure 2). The gel was found to be composed of laminated aggregate of thin layers, which were tens of nm~μm wide, and tens of μm long. The gel bands were observed to be folded, stacked, and fused together. These thin-layer agglomerates are loosened in the solution–dispersion state and are thought to exhibit a network structure similar to the “house of cards” seen in clay minerals [7,8], indicating a gel state. As a result of the fusion, folding and deformation of these strips, many wrinkles were observed on the surface of the **PR-U-3** 5.0 wt.% dried sample, which is a xerogel obtained by drying DMF gel, and therefore the stacking and fusion were observed during the drying process; this SEM observation is inferred to reflect the fact that the **PR-U-3** 5.0 wt.% gel was composed of reticulated material. The formation of a network of such bands has also been observed in SEM images of other molecular gel xerogels obtained by a polymer gelator [23].

**Figure 3:**
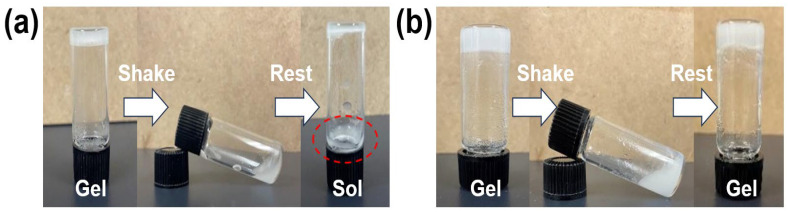
Thixotropic behaviors of the **PR-U-3** DMF molecular gels: (**a**) 5.0 wt.% **PR-U-3** and (**b**) 10.0 wt.% **PR-U-3**.

Next, the thixotropic properties of the 5.0 wt.% **PR-U-3** and 10.0 wt.% **PR-U-3** gels were evaluated by the vial-inversion method, and the results revealed that the thixotropic properties of the 5.0 wt.% **PR-U-3** gel (Figure 3a) were not recovered. The sample failed to return to its gel state, remaining in the liquid state even after its disintegration by an external mechanical force produced by a vortex mixer, as well as standing for 1 min. However, the 10.0 wt.% **PR-U-3** gel (Figure 3b) was thixotropic, as it regenerated into a gel after being allowed to stand for 30 min following its disintegration into a sol state; the regenerated gel did not flow downward when the vial was inverted. Thus, the thixotropic property of the 10.0 wt.% **PR-U-3** gel was qualitatively confirmed by vial inversion.

**Figure 4:**
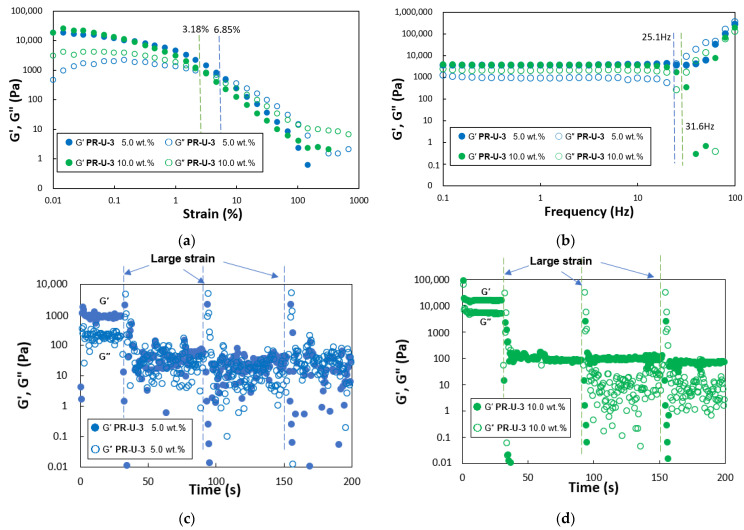
Dynamic rheological properties of the **PR-U-3** DMF molecular gels: (**a**) strain dispersion and (**b**) frequency dispersion measurements. Thixotropic evaluations of the (**c**) 5.0 wt.% **PR-U-3** and (**d**) 10.0 wt.% **PR-U-3** DMF gels.

To evaluate the mechanical properties of the DMF gels, the prepared 5.0 wt.% **PR-U-3** and 10.0 wt.% **PR-U-3** gels were subjected to dynamic viscoelasticity evaluations (strain dispersion measurement, frequency dispersion measurement, and thixotropy evaluation) (Figure 4). First, the results of the strain dispersion measurements of the two gel samples (Figure 4a) revealed that as the G′ and G″ values represent the storage and loss moduli, respectively; we assumed that the measured sample transitioned from a gel to a sol at the intersection of the G′ and G″ values, where the state of G′ > G″ (gel) to G′ < G″ (sol) was reached [36,37]. Figure 4a shows that the 5.0 wt.% **PR-U-3** and 10.0 wt.% **PR-U-3** samples were in the gel state before the measurements. Thereafter, the 5.0 wt.% **PR-U-3** and 10.0 wt.% **PR-U-3** samples transitioned from gel to sol states under strains of 6.9% and 3.2%, respectively. These findings revealed that the 10.0 wt.% **PR-U-3** with a higher gelator concentration transitioned from a gel to a sol state at a lower strain, indicating the formation of a soft gel that transitions into a sol state at a lower strain by increasing the amount of the gelator, **PR-U-3**. Generally, the higher the material density, the stiffer the gel becomes, although the decrease in the gel–sol transition strain with this increasing gelator concentration may be due to the **PR** structure in the gelator.

Next, the results of the frequency dispersion measurements of the two gel samples are shown in Figure 4b. The 5.0 wt.% **PR-U-3** and 10.0 wt.% **PR-U-3** samples exhibited G′ > G″ at the beginning of the measurement, indicating that both samples proceeded from the gel state. Next, they exhibited G′ < G″ at frequencies of 25.1 and 31.6 Hz for 5.0 wt.% **PR-U-3** and 10.0 wt.% **PR-U-3**, respectively, indicating that the samples transitioned from gel to sol states at this point. The transitioning of the 10.0 wt.% **PR-U-3** from gel to sol occurred at a higher frequency than that of the 5.0 wt.% **PR-U-3**. This was attributable to the improved stability of the gel state, following the increase in the concentration of the gelator **PR-U-3**. The different trends from those of the strain-dispersed results reported earlier indicate that the molecular gel that was formed from the **PR** structure gelator exhibited different properties from general molecular gels, which exhibit the same gel trends for the strain and frequency dispersions.

Figure 4c shows the results of the thixotropic evaluation of both gel types. The 5.0 wt.% **PR-U-3** gel exhibited G′ < G″ at 30, 90, and 150 s after applying a large strain. This indicated that the gel state transitioned to a sol state at that time. Even after discontinuing the application of the large strain, the G′ > G″ state was not stably recovered, and the G′ and G″ values varied. Thus, no clear thixotropic property was observed in the 5.0 wt.% **PR-U-3** gel. At the beginning of the measurements, the G′ and G″ values were lower than those of the strain dispersion and frequency dispersion measurements, and this is probably because the 5.0 wt.% **PR-U-3** gel was a very loose one, which disintegrated when scooped with a spoon. Thus, it exhibited an unstable gel state. The 10.0 wt.% **PR-U-3** gel exhibited a G′ < G″ state when a large strain was applied. Thereafter, it recovered the G′ > G″ after the applied large strain was removed, and it became slightly more stable compared to the 5.0 wt.% **PR-U-3** gel. This result indicates that the 10.0 wt.% **PR-U-3** gel exhibited pseudo-thixotropic properties, as the gel was mechanically disintegrated by the applied strain and transitioned to a sol state, soon returning to the gel state [22]. The result also confirmed that the pseudo-thixotropic property was observed repeatedly by alternating small and large strains. This confirmation of the pseudo-thixotropic properties of the 10.0 wt.% **PR-U-3** gel agrees with the results of the qualitative thixotropic evaluation using vial inversion, as described above. As the “house of cards” structure network of nanodisk-shaped synthetic silicate laponite exhibits thixotropic properties in dispersed solution [38,39], it is likely that this gel system, a thin-layer gelator aggregate observed in the SEM images, exhibits thixotropic properties. However, the fact that the vial test showed thixotropy but the rheometry showed a dispersion of data that could be called pseudo-thixotropy means that the recovery after breakdown or stability after recovery of the network structure of this system is not sufficient. However, since recovery behavior after fracture was observed, it was described as pseudo-“thixotropic” behavior.

**Figure 5:**
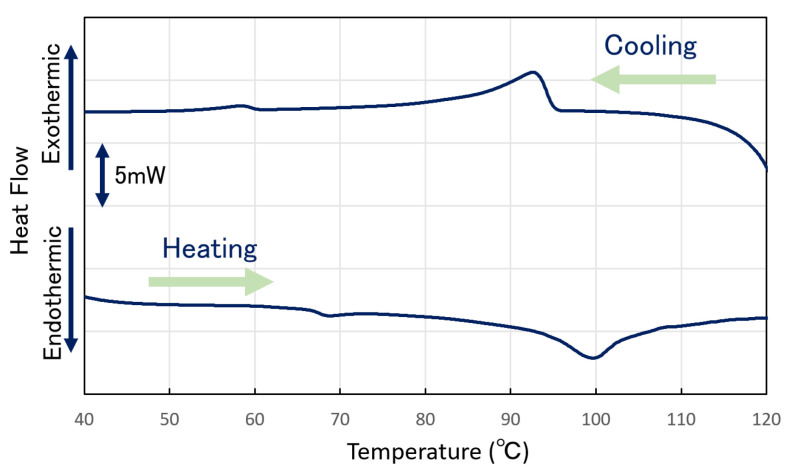
DSC curves of the **PR-U-3** 10.0 wt.% DMF gel (heating and cooling rate is 10 °C/min).

The thermal behavior of the 10.0 wt.% **PR-U-3** DMF gel was investigated by differential scanning calorimetry (DSC). Regarding the results (Figure 5), the peak at 86.5 °C was attributed to the change from the gel to sol states of the sample. In the temperature reduction process, we observed heat release at 95.3 °C and 60.5 °C. The peaks at 95.3 °C and 60.5 °C were attributed to the change from the gel to sol states of the sample. The peak from 95.3 °C corresponded to the change from the sol to gel states of the sample. The peaks at 65.3 °C and 60.5 °C during the temperature increase and decrease, respectively, may be due to the rotaxane structure of the sample, such as the rotational and translational motions of α-CD. Here, the calorific values of each peak are as follows: 0.64 mJ/mg (65.3 °C) and 12.2 mJ/mg (86.5 °C) for the temperature increasing process and 0.71 mJ/mg (60.5 °C) and 12.2 mJ/mg (95.3 °C) for the temperature decreasing process; as the corresponding absorption and endothermic peaks were almost the same, they represent the heat in and out of the corresponding processes [1]. The first peak from the low temperature side was considered the activation and freezing of the thermal motion of α-CD, and the second peak corresponded to the sol–gel transition behavior of the **PR** gelator.

Finally, to apply the polymer organogelator **PR-U-3** to the formation of the DMF gels, we prepared polymer composites by incorporating the gelators into polymer materials. In this study, micrometer order band-like structures, formed by **PR-U-3**, were composited with polymer materials in a DMF solution to improve the mechanical strength of the composites through the filler effect [40,41]. Next, rubber bands, which were made of natural rubber, were selected as the polymer material, and **PR-U-3** was incorporated into the rubber bands by incorporating the rubber bands into the above DMF gel preparation process (this can be achieved by immersing the rubber bands in the DMF sol solution), followed by the evaluation of the mechanical properties by tensile testing. To demonstrate the effect of the gelator concentration on the mechanical properties, the **PR-U-3** concentrations of DMF were varied at 0.1 wt.%, 1.0 wt.%, and 5.0 wt.%. After immersing the rubber bands in the DMF gelator solution, the gelator was heated at 110 °C and dissolved for 30 min, after which it was allowed to cool. Next, the rubber bands, as well as the remaining solution or gel on the surface, were removed. Further, the rubber band samples with the incorporated gelator were vacuum-heated and dried. The weight ratios of **PR-U-3** in the rubber bands (based on the rubber bands before the incorporation), as calculated from the weight change in the rubber band samples before and after the incorporation of the gelling agent, were 0.7 wt.%, 0.7 wt.%, and 2.9 wt.% for the 0.1 wt.% **PR-U-3**, 1.0 wt.% **PR-U-3**, and 5.0 wt.% **PR-U-3** DMF solutions, respectively, as averaged using five samples. At 5.0 wt.%, the DMF solution yielded a weight ratio of 2.9 wt.%. **PR-U-3** was incorporated into some samples above the solution concentration, and this might be due to the **PR-U-3** concentration of the surface layer of the rubber bands via its interaction with polyisoprene, the raw material of the rubber bands.

**Figure 6:**
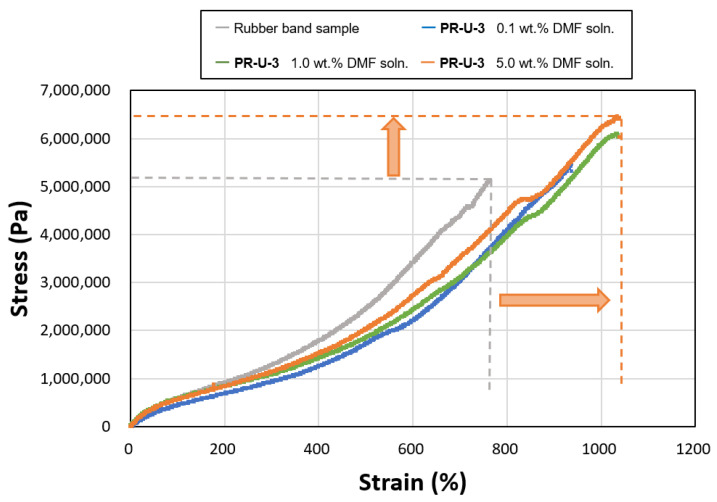
Tensile test results for the **PR-U-3**-composited rubber bands.

Figure 6 shows the tensile test results for each sample. The DMF-immersed rubber band sample, which was heated and vacuum-dried without the gelator, exhibited an elongation at break of 763.2% and stress at break of 5.1 MPa. The rubber band samples immersed in the 0.1 wt.% **PR-U-3** DMF solution exhibited an elongation at break of 937.2% and stress at break of 5.3 MPa. The rubber band samples immersed in the 1.0 wt.% **PR-U-3** DMF solution exhibited an elongation at break of 1035.3% and stress at break of 6.0 MPa. The rubber band sample immersed in the 5.0 wt.% **PR-U-3** DMF solution, which has the highest gelator concentration, exhibited an elongation at break of 1038.4% and stress at break of 6.0 MPa. We observed that the incorporation of the **PR-U-3**, a gelling agent exhibiting a **PR** structure, increased the elongation at break and stress at break of the rubber bands. The breaking strain and stress results of the rubber band samples immersed in 1.0 wt.% **PR-U-3** and 5.0 wt.% **PR-U-3** DMF solutions were not significantly different, although the breaking strain and stress increased as the concentration of the **PR-U-3** solution increased. This confirmed that the addition of **PR-U-3** did not increase the strength of the rubber bands and that the rubber bands had limits. In conclusion, the mechanical properties of the polymer composite obtained by introducing **PR-U-3** into the polymer material were enhanced.

**Figure 7:**
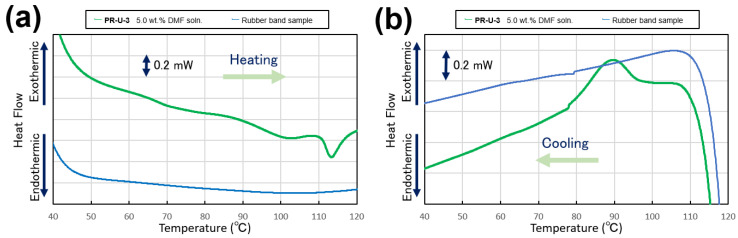
DSC curves of the 5.0 wt.% **PR-U-3**-composited rubber (green lines) and non-composited rubber (blue lines) at heating and cooling rates of 10 °C/min in each case: (**a**) heating and (**b**) cooling processes.

The DSC results of the rubber bands, following their pretreatment (not composited) and immersion in the 5.0 wt.% **PR-U-3** DMF solution, are shown in Figure 7. Similarly, in the temperature reduction process, several peaks were observed only in the rubber bands that were immersed in the 5.0 wt.% **PR-U-3** DMF solution. As these peaks correspond to the sol–gel transition behavior of the **PR** gelator identified in the thermal analysis of the organogelator in this study, we assumed that the **PR-U-3** underwent the same sol–gel phase transition in the rubber bands as when it was a gel. Put differently, we believe that **PR-U-3** could be incorporated into rubber bands.

**Figure 8:**
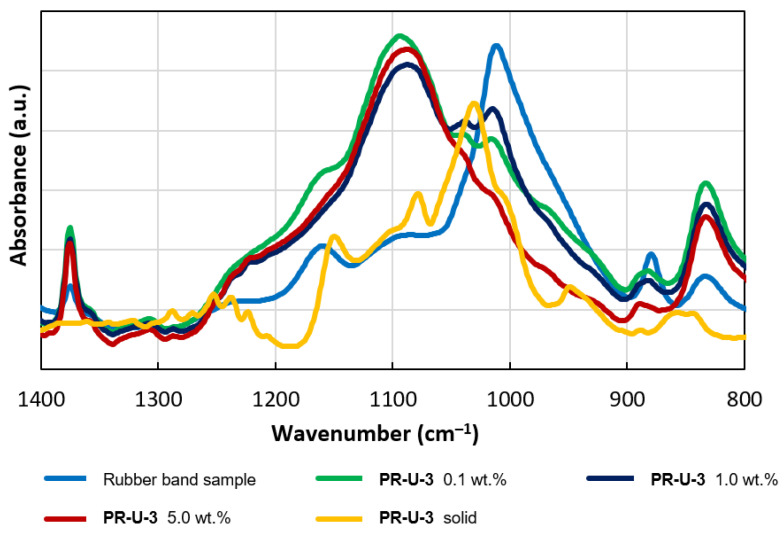
ATR–FTIR spectra of the **PR-U-3**-composited rubbers.

The attenuate total reflectance for the Fourier transform infrared spectroscopy (ATR–FTIR) results for the **PR-U-3**, pretreated rubber bands (non-composited), and rubber bands immersed in the 0.1 wt.%, 1.0 wt.%, and 5.0 wt.% **PR-U-3** DMF solution are shown in Figure 8. The strong absorption at 1007 cm^−1^ and medium absorption at 881 cm^−1^, which were due to the stretch vibration of C–C, of polyisoprene and the out-of-plane angular bending vibration of alkene C–H in the main chain, respectively [42,43], were observed in the rubber bands after the pretreatment with 0 wt.% **PR-U-3**. By blending **PR-U-3** with the rubber bands, we observed an absorption band at approximately 1080 cm^−1^. This band was not observed in the pretreated (non-composited) rubber bands or **PR-U-3** alone. Additionally, the alkene C–H peak tended to decrease as the concentration of **PR-U-3** increased. Although the observed absorption band was unclear owing to complexation, it was assumed to be a new absorption band due to the intermolecular interaction between the rubber bands and **PR-U**. We inferred that the interaction between the rubber and the gelator filler is related to the improvement in mechanical properties in the tensile test described above, i.e., the filler effect [40,41].

## 3. Conclusions

In this study, we synthesized **PR-U-3**, a polymer organogelator exhibiting an octadecyl carbamate-modified **PR** structure, and evaluated its gelation properties. Further, we employed it for the fabrication of polymer composites. We observed that **PR-U-3** was a polymer organogelator that could facilitate the gelling of DMF, a solvent that can dissolve polymers and salts. Additionally, the DMF molecular gels exhibited pseudo-thixotropic properties when the concentration of **PR-U-3** was 10.0 wt.%. This suggests that this DMF molecular gel containing inorganic salts is applicable as a gel electrolyte. Furthermore, polymer composites containing the polymeric organogelator were fabricated with rubber bands, and the composite samples with the incorporated organogelator exhibited higher mechanical properties than those without the organogelator, as the elongation and stress at break increased, indicating the fabrication of a new polymer composite material (a gelator/polymer material). As the new polymeric organogelator could be composited with polymer materials and improve their polymeric properties, it could be incorporated into polymer to improve the mechanical properties of polymeric materials.

## 4. Materials and Methods

Poly(ethylene glycol) (PEG, molecular weight: 35,000) was purchased from Sigma-Aldrich Japan (Merck KGaA, Darmstadt, Germany) and used as received. Other reagents except organic solvents were purchased from Tokyo Chemical Industry Co., Ltd., Tokyo, Japan, and were not further purified. The organic solvents used were purchased from Wako Pure Chemical Industries, Ltd., Tokyo, Japan, and were not further purified.

To obtain hydroxypropylated polyrotaxane (**HP-PR**), we first synthesized adamantyl-capped polyrotaxane composed of PEG and α-CD (**Ad-capped-PR**), which was obtained by the method described in the literature [35]. According to the literature for **Ad-capped-PR**, the inclusion rate of CD into the PEG chain is 22–25% based on the ethylene oxide moiety (ethylene oxide and CD can form a 1:1 inclusion complex) [35]. **HP-PR** was then synthesized by hydroxypropylation of the hydroxyl group at position 6 of α-CD of this **Ad-capped-PR** with propylene oxide according to the method described in the literature [34]. The ^1^H-NMR spectrum of the **HP-PR** showed that the number of hydroxypropyl groups in the **HP-PR** was found to be 39% of the total number of hydroxyl groups at position 6 of α-CD [44].

Octadecy-modified polyrotaxanes with carbamate linkages in the polymer side chain (**PR-U**) were synthesized by chemical reaction of **HP-PR** with octadecyl isocyanate at the hydroxyl group of α-CD in DMF in the presence of a tin catalyst. Different amounts of octadecyl isocyanate were synthesized. In the general procedure, dibutyltin dilaurate (DTDL), 500 mg **HP-PR**, and 4 mL DMF were added to a 50 mL Nas flask and stirred at room temperature to form a uniform solution after nitrogen displacement. Then, 4 mL of octadecyl isocyanate (C18I) in DMF was added to the **HR-PR** mixture at room temperature, and the nitrogen was replaced. The mixed solution was placed in a water bath at 60 °C and heated and stirred for 90 min. After the reaction, the mixed solution was added to diethyl ether and reprecipitated, and then the target product was redissolved in DMF, added to diethyl ether, and reprecipitated again. After allowing the reprecipitated solution to stand, the precipitate was filtered and vacuum-heated, and dried (70 °C, 5 h) to obtain the white solid object. The percentage of C18I incorporated into **HP-PR** of reactive products (C18I ratio) was calculated from the ratio of integrals in the ^1^H-NMR measurements with results of elemental analysis. The reagents’ weights, yields, and calculated results are shown below. Nuclear magnetic resonance spectroscopy was used to identify the molecular structure of the products, and ^1^H-NMR was measured by the FT-NMR system JNM-ECZ400 (JEOL Ltd., Tokyo, Japan). The results of elemental analysis for the polymers were obtained with YANACO CHN CORDER MT-5 (organic trace element analyzer, YANACO technical science, Co., Ltd., Tokyo, Japan). The results are as follows: 

**PR-U-1**: C18I 165 mg, DTDL 15 mg, yield 535.1 mg; C18I ratio: 16%. ^1^H-NMR (DMSO-d_6_, δ in ppm) 7.91 (s, 1H, –NHCOO–), 5.34–6.10 (m, –OH), 4.76 (s (br), –CH (6-position of α-CD CH)), 4.45–4.49 (m, –OH), 3.26–3.68 (m, –CH_2_, –CH–, –OH), 1.13–1.19 (m, 32H, octadecyl –CH_2_–), 0.98 (m, hydroxypropyl –CHCH(OH)*CH*_3_), 0.81 (t, J = 6.40 Hz, 3H, octadecyl-CH_2_*CH*_3_). Elemental analysis result: Found: C, 53.76; H, 6.68; N, 1.50.

**PR-U-2**: C18I 412 mg, DTDL 37 mg, yield 667.5 mg; C18I ratio: 37%. ^1^H-NMR (DMSO-d_6_, δ in ppm) 7.91 (s, 1H, –NHCOO–), 5.27–6.08 (m, –OH), 4.76 (s (br), –CH (6-position of α-CD CH)), 4.44–4.50 (m, –OH), 3.25–3.68 (m, –CH_2_, –CH–, –OH), 1.13–1.19 (m, 32H, octadecyl –CH_2_–), 0.97 (m, hydroxypropyl-CHCH(OH)*CH*_3_), 0.81 (t, J = 6.40 Hz, 3H, octadecyl-CH_2_*CH*_3_). Elemental analysis result: Found: C, 61.90; H, 10.29; N, 2.80.

**PR-U-3**: C18I 659 mg, DTDL 59 mg, yield 973.8 mg; C18I ratio: 44%. ^1^H-NMR (DMSO-d_6_, δ in ppm) 7.95 (s, 1H, –NHCOO–), 5.41–5.98 (m, –OH), 4.75–4.86 (m, –CH (6-position of α-CD CH)), 4.45–4.47 (m, –OH), 3.68–3.26 (m, –CH_2_, –CH–, –OH), 1.23–1.36 (m, 32H, octadecyl –CH_2_–), 1.03 (m, hydroxypropyl -CHCH(OH)*CH*_3_), 0.84 (t, J = 6.40 Hz, 3H, octadecyl-CH_2_*CH_3_*). Elemental analysis result: Found: C, 64.45; H, 10.82; N, 3.32.

The inversion method using vials for gelation evaluation is as follows: the **HP-PR** or **PR-U** and a predetermined amount of DMF were placed in a vial, heated in a dry bath at 110 °C for 30 min, and allowed to stand at room temperature for 30 min. Then, the gelation ability was evaluated by inverting the vial and checking whether the mixed solution dripped. The qualitative evaluation method for thixotropic properties was performed by the inverted method using one vial. In this method, first, the gel prepared in the mighty vial is shaken with a vortex mixer for several seconds to break it down to a sol state mechanically. Next, the resulting sol is allowed to stand at room temperature for several minutes, and then the vial is inverted to confirm that it has returned to a gel state by visual observation. If the mixture does not drip when the vial is inverted, it is considered to have gelled back, and the gel is considered to have thixotropic properties.

For the scanning electron microscope (SEM) observation, samples of **PR-U-3** 5.0 wt.% dry gel were vacuum-dried at room temperature for 3 h and at 70 °C for 3 h. The instrument used in this study was a field emission scanning electron microscope JSM-6700FN (JEOL Ltd., Tokyo, Japan), which was used at an acceleration voltage of 1 keV to minimize sample damage after conductive coating with a PtAu ion sputter (E-1045, Hitachi High-Technologies Corporation, Tokyo, Japan).

Dynamic viscoelasticity measurements of gels were performed using an MCR 302e Modular Compact Rheometer (Anton Paar Japan K.K., Tokyo, Japan). An 8 mm diameter parallel plate was used, and the sample was placed between the fixture and the stage with a gap of 0.5 mm. Any protruding gel was wiped off and measurements were performed at a measurement temperature of 25 °C. For the strain dispersion measurement, the frequency was fixed at 1 Hz and the strain was varied from 0.01 to 1000%. For the frequency dispersion measurement, the strain was fixed at 1% and the frequency was varied from 0.1 to 100 Hz. For the thixotropic evaluation, a high shear rate (1000 s^−1^) was applied to the sample at 0.1% strain and 1 Hz frequency, 30 s after the start and every 60 s thereafter, and the change in the modulus over time and the structural recovery of the sample were observed [21].

The thermophysical properties of the composites were measured using a high-sensitivity differential scanning calorimeter DSC7000 (Hitachi High-Tech Corporation, Tokyo, Japan) by increasing and decreasing the temperature from 30 °C to 120 °C to −10 °C at a temperature increase of 10 °C/min under a nitrogen atmosphere (sample container: sealed Ag container, reference sample: empty sealed Ag container).

The composite rubber band samples were tensile-tested using a tabletop universal testing machine MCT-2150 (A&D Company, Limited, Tokyo, Japan). The rubber band samples were cut to a sample length of 3 cm, and the cross-sectional areas were measured with a digital multimeter. The sample was fixed so that the length between the jigs of the testing machine was 1 cm, pulled at a speed of 10 mm/min, and the strain and stress when the sample broke were recorded.

Molecular interactions in composites were evaluated by infrared spectroscopy using the ATR-FTIR method, which combines an FTIR6600 spectrometer (JASCO Corporation, Tokyo, Japan) and a single bounce diamond attenuated total reflectance unit.

## Data Availability

Not applicable.
